# Production of recombinant soluble dimeric C-type lectin-like receptors of rat natural killer cells

**DOI:** 10.1038/s41598-019-52114-8

**Published:** 2019-11-28

**Authors:** Ondřej Vaněk, Petra Celadova, Ondřej Skořepa, Jan Bláha, Barbora Kalousková, Anna Dvorská, Edita Poláchová, Helena Pucholtová, Daniel Kavan, Petr Pompach, Kateřina Hofbauerová, Vladimír Kopecký, Aruz Mesci, Sebastian Voigt, James R. Carlyle

**Affiliations:** 1 Department of Biochemistry, Faculty of Science, Charles University, Hlavova 2030/8, 12840 Prague, Czech Republic; 2 Institute of Microbiology, The Czech Academy of Sciences, Vídeňská 1083, 14220 Prague, Czech Republic; 3 Institute of Physics, Faculty of Mathematics and Physics, Charles University, Ke Karlovu 5, 12116 Prague, Czech Republic; 4 Department of Immunology, University of Toronto, 1 King’s College Circle, M5S 1A8 Toronto, ON Canada; 5 Department of Infectious Diseases, Robert Koch Institute, Seestraße 10, 13353 Berlin, Germany; 6 Present Address: EMBL Hamburg, c/o DESY, Building 25A, Notkestraße 85, 22603 Hamburg, Germany

**Keywords:** Glycoproteins, Recombinant protein therapy, Expression systems, Transfection, NK cells

## Abstract

Working at the border between innate and adaptive immunity, natural killer (NK) cells play a key role in the immune system by protecting healthy cells and by eliminating malignantly transformed, stressed or virally infected cells. NK cell recognition of a target cell is mediated by a receptor “zipper” consisting of various activating and inhibitory receptors, including C-type lectin-like receptors. Among this major group of receptors, two of the largest rodent receptor families are the NKR-P1 and the Clr receptor families. Although these families have been shown to encode receptor-ligand pairs involved in MHC-independent self-nonself discrimination and are a target for immune evasion by tumour cells and viruses, structural mechanisms of their mutual recognition remain less well characterized. Therefore, we developed a non-viral eukaryotic expression system based on transient transfection of suspension-adapted human embryonic kidney 293 cells to produce soluble native disulphide dimers of NK cell C-type lectin-like receptor ectodomains. The expression system was optimized using green fluorescent protein and secreted alkaline phosphatase, easily quantifiable markers of recombinant protein production. We describe an application of this approach to the recombinant protein production and characterization of native rat NKR-P1B and Clr-11 proteins suitable for further structural and functional studies.

## Introduction

Natural killer (NK) cells are innate immune lymphocytes capable of recognizing and destroying a wide variety of target cells, including transformed, infected, transplanted, antibody-coated, and stressed cells^1^. In contrast to T or B cells, NK cells do not express a single dominant activation receptor on their surface. Instead, the functions of these immune effector cells are regulated by a high number of receptors that generate either inhibitory or activation signals through the large “receptor zipper”^2^. These receptors belong to two major protein families similar to members of the immunoglobulin or C-type lectin superfamilies^3^. Inhibitory C-type lectin receptors, such as Ly-49 proteins or CD94/NKG2A heterodimers, recognize MHC class I glycoproteins on the surface of healthy cells and efficiently block the natural killing of these cells. However, in pathological states, target cells often lose inhibitory “self” ligands, leading to enhanced cytotoxicity via NK cell disinhibition. This mode of detection has been termed “missing-self” recognition^4^. Conversely, stressed, transformed or infected cells often overexpress some proteins that are usually in low abundance, on healthy cells. These proteins might be recognized by activating receptors (induced-self-recognition), as in the case of the stress-induced MHC class I-like molecules MICA and MICB, which are recognized by the NKG2D activation C-type lectin-like receptor^5^.

In addition to Ly-49, the NKR-P1 (CD161) receptor family, which is the second largest group of receptors encoded by distinct but closely related genes of the NK gene complex, may generate both activation and inhibitory signals in NK cells. This protein family was first identified on the surface of rat natural killer cells using a specific monoclonal antibody 3.2.3^6^. Subsequent studies showed that NKR-P1 (CD161) receptors consist of homodimeric type II transmembrane C-type lectin-like proteins primarily found on NK, NKT, and activated CD8^+^ T cells^7^. Genes encoding the NKR-P1 family (designated *Klrb1*) appear to be conserved amongst birds, rodents, humans, and other mammals, suggesting that the gene products play a key role in innate immunity across species boundaries^8^. The NKR-P1 receptors consist of N-terminal peptide motifs involved in receptor signalling, a single transmembrane domain, an extended stalk region that includes the putative dimerization cysteine(s), and a large C-terminal ligand binding domain similar to the carbohydrate-recognition domain of C-type lectins^9^.

The discovery that physiological ligands of at least some NKR-P1 receptors belong to a family of related C-type lectin-like receptors, the C-type lectin-related (Clr) proteins, which are encoded by a family of *Clec2* genes interspersed among the *Klrb1* genes themselves, was a breakthrough in understanding NKR-P1 function^10,11^. These studies have shown that mouse NKR-P1B recognizes Clr-b and that transfected cells expressing Clr-b are partly protected from lysis by NK cells, thus suggesting that NKR-P1B:Clr-b recognition is a novel form of missing-self recognition designed to monitor the cellular levels of Clr-b. Accordingly, genetic linkage of the *Clec2* and *Klrb1* genes highlighted the importance of this system as a particularly unique self/non-self discrimination tool because the “self” ligand is always coinherited with its cognate receptor^7,12^. These findings established a new paradigm of lectin-like receptors that interact with other lectin-like proteins rather than with carbohydrates, although the role of receptor and ligand glycosylation in these interactions remains unknown.

In addition to mice, this MHC-independent self-recognition system is conserved at least in rats^13^ and humans^14,15^. Similarly to MHC-I molecules, this self-recognition system is also subject to viral and tumour evasion of innate and adaptive immunity. In humans, the LLT1 receptor, an orthologue of rodent Clr proteins, is upregulated in glioblastoma^16^, in prostate cancer^17^ and in B-cell non-Hodgkin’s lymphoma^18^, in which the receptor mediates immune escape and contributes to the immunosuppressive properties of tumour cells. Conversely, rat cytomegalovirus encodes a protein named RCTL that closely resembles rat Clr-11 (a homolog of mouse Clr-b). Viral infection stimulates Clr-11 loss, which is rapidly counteracted by RCTL surface expression upregulation. RCTL inhibits NK killing of infected cells via direct interaction with NKR-P1B. Thus, RCTL functions as a decoy ligand to subvert NKR-P1B mediated missing-self recognition by NK cells^19^. Interestingly, this subversion is strain-dependent: the NKR-P1B receptor from the WAG rat strain is susceptible to RCTL binding, whereas the NKR-P1B receptor from the SD rat strain is less susceptible, thereby overcoming this decoy inhibition signal. The allelic divergence of rodent NKR-P1 receptors suggests that host genomes are evolving under selection pressure to avert this viral evasion strategy^7,20^. Similarly, in mice, Clr-b loss was observed upon murine cytomegalovirus (MCMV) infection^21^. Furthermore, the MCMV-encoded immunoevasin m12 was observed to engage the inhibitory NKR-P1B receptor, thus subverting the NKR-P1B:Clr-b immune axis. However, a similar host-pathogen evolutionary interplay is revealed by the engagement of some of the m12 alleles through the activating NKR-P1A/C receptors that avert the MCMV decoy strategy^22^. The mouse Clr-b ligand is also a very sensitive marker of cell health that is rapidly downregulated during chemotherapy-induced genotoxic and cellular stress^23^ or poxvirus infection^24 or oncogenesis25^. Concomitantly, recent studies showed that NKR-P1B:Clr-b missing-self recognition plays a key and non-redundant role in bone marrow transplantation^26,27^ and cancer immunosurveillance^25^ in a mouse models.

Although the structures of a few mouse NKR-P1 and Clr proteins, as well as the structure of the mouse NKR-P1B:m12 complex, were published^22,28,29^, only limited structural data on the NKR-P1:Clr receptor complex are available yet. In some cases, the preparation of soluble C-type lectin-like receptor domains by recombinant expression in *E*. *coli* followed by *in vitro* refolding might be relatively easy^28,29^. However, this strategy also has several disadvantages. The refolding yields are often too low for structural studies, and the number of cysteine residues present in the expression constructs is usually kept as low as possible, leading to monomeric recombinant proteins (or non-covalent dimers, at best). However, native C-type lectin-like NK receptors are often homodimers linked by one or several disulphide bridges^30^, and stable dimer formation might also be a prerequisite for complex formation. Moreover, the role of glycosylation in NKR-P1:Clr recognition could not be ascertained using bacterially expressed proteins.

In this report, we describe a eukaryotic expression system based on transient transfection of a human embryonic kidney 293 (HEK293) cell line to produce native dimeric NK cell C-type lectin-like receptors for structural and functional studies. In contrast to stable transfection and cell line generation, transient transfection offers a quick modularity of the expression construct regarding the purification or visualization tag(s), if necessary. Simultaneously, by using cost-effective transfection reagents, affordable cell culture media and strong expression vectors, milligram amounts of recombinant proteins can be generated within days at only moderate costs and production equipment requirements^31,32^. Moreover, successful selenomethionine incorporation and N-linked glycosylation control, which are important for structural biology, particularly for protein crystallography, were shown in HEK293 cell lines^32,33^. Here, we report the successful application of this technique to the recombinant production of rat C-type lectin-like NK cell receptors.

## Results

### Optimized transient transfection of suspension-adapted HEK293T cells

Transient transfection of adherent HEK293T cell lines with several pHLsec plasmids has been successfully used for recombinant protein expression^32^. However, in our laboratory, the growth of high cell quantities in expanded surface roller bottles was rather difficult due to uneven cell attachment to the surface of the culture bottles. Moreover, the expansion of adherent culture prior to large-scale transient transfection using this method was a time-consuming, labour-intensive, and material-demanding procedure. Therefore, we decided to switch to suspension culture, which is affordable (glass bottles can be autoclaved and reused easily), scalable in a range from a few millilitres to hundreds of litres and may yield potentially higher volumetric productivity.

**Figure 1:**
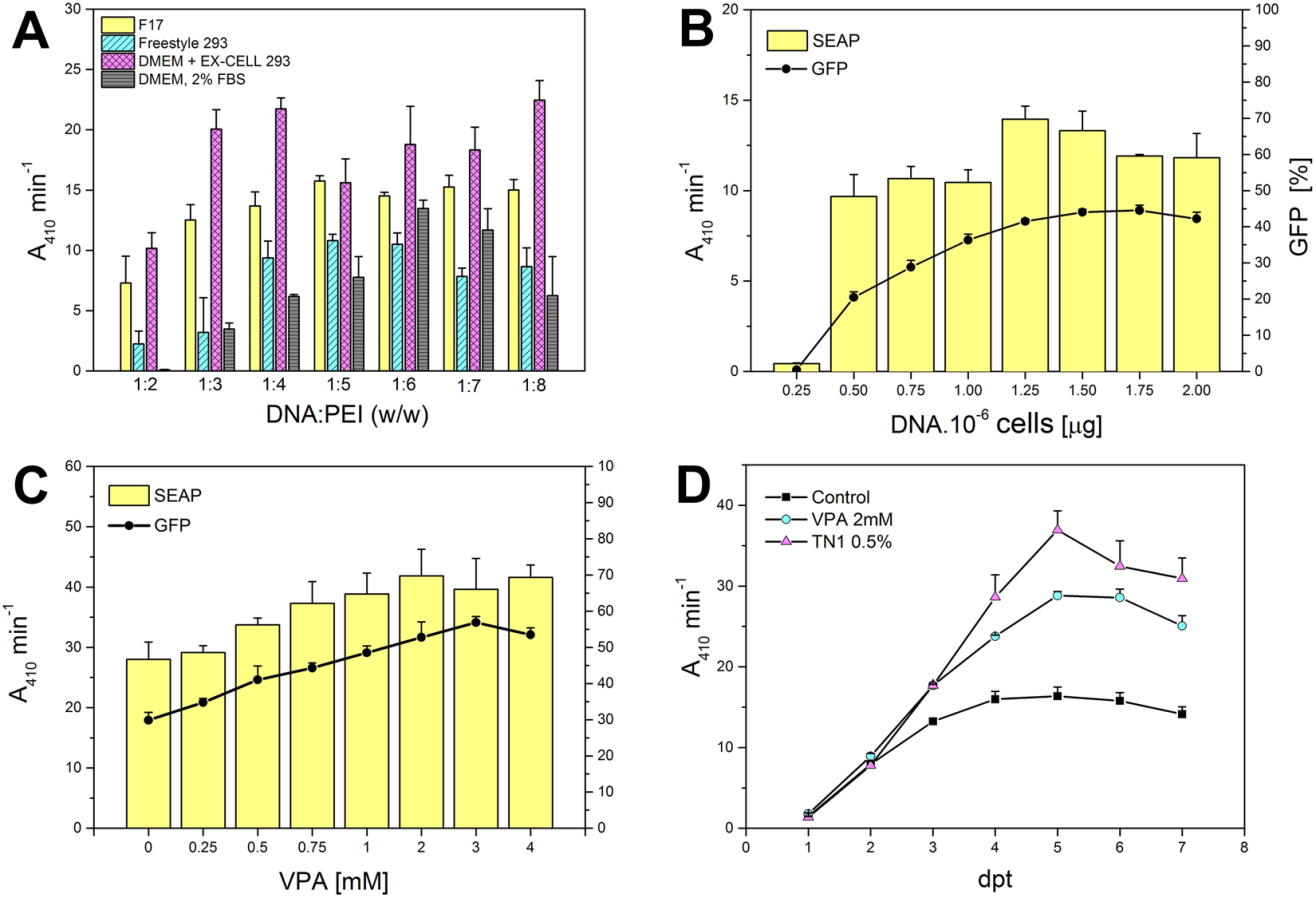
Optimization of transient transfection of suspension adapted HEK293T cells. (**A**) Effect of the DNA-to-PEI ratio on SEAP production in different transfection and production media. (**B**) Effect of the amount of DNA added to the cells. (**C**) Enhancement of transient expression by valproic acid addition at 3 hours post-transfection; transfections were performed in 24-well plates, as described in Materials and Methods, SEAP activity and transfection efficiency were determined 3 days post-transfection (dpt) by measuring the pNPP hydrolysis rate and by flow cytometry (GFP positive viable cells), respectively. (**D**) Transient transfection expression levels as a function of time; SEAP activity was measured each dpt in a control culture, in a culture supplemented with 2 mM VPA and in a culture fed at 2 dpt with 0.5% TN1 hydrolysate; transfections were performed in a 12-well plate, as described in the Materials and Methods section.

An aliquot of 5 × 10^6^ adherent HEK293T cells was thawed and transferred directly into 10 ml of EX-CELL 293 serum-free media, in a 10-cm culture dish. Small floating clumps of cells resumed growth one day later. The culture was pipette-resuspended and split into fresh medium every three days, and we considered the cells fully adapted to serum-free conditions after six splits. Then, we transferred the cell suspension into a square-shaped glass bottle, which was placed on an orbital shaker inside the incubator. Cells resumed growth one day later and grew with a doubling time of approximately 24 h. The EX-CELL 293 medium supports HEK293T high-density cultivation (up to 6 × 10^6^/ml with no apparent loss of viability) and is also suitable for freezing the cells (up to 5 × 10^7^/ml with 5–10% DMSO tested). In contrast, EX-CELL 293 is unsuitable for polyethylenimine (PEI) mediated transfection, most likely due to the presence of negatively charged additives (such as heparin, often used to dissociate cell clumps), which disrupt DNA-PEI transfection polyplexes. We screened several other media for their transfection and production properties using secreted alkaline phosphatase (SEAP) and green fluorescent protein (GFP) as easily quantifiable reporter proteins to monitor secreted protein production and transfection efficiency, respectively. A small volume of cell suspension was transfected with a 19:1 (w/w) mixture of SEAP and GFP expression plasmids, and SEAP activity and transfection efficiency were measured 72 hours later. From the two commercial media tested, F17 medium allows the use of lower DNA:PEI ratios, thus minimizing the risk of potential cytotoxic effects of high PEI concentrations (Fig. 1A). Because the original adherent HEK293T cells were easily transfected in standard DMEM medium supplemented with 2% FBS, we assessed whether these conditions could be modified for suspension conditions. By omitting calcium chloride from the DMEM formulation and by supplementing it with 0.1% Pluronic F-68 instead, we were able to maintain the cells in suspension, although the addition of 2% FBS caused cell aggregation. Conversely, the highest SEAP activities were achieved when transfection was performed in half the desired culture volume of calcium-free DMEM, which was completed with EX-CELL 293 to the full volume 3 hours post-transfection (Fig. 1A). All subsequent transfections were performed in calcium-free DMEM/EX-CELL 293 at a 1:4 (w/w) DNA:PEI ratio.

To maximize the expression yield, we assessed the effect of the DNA amount added to the cells. As shown in Fig. 1B, the amount of DNA necessary for sufficient transfection efficiency and SEAP expression could not be reduced below 1 μg/10^6^ cells under these conditions. However, both transfection efficiency and SEAP expression almost doubled when valproic acid was added 3 hours after transfection (Fig. 1C). Valproic acid is a histone deacetylase inhibitor reported to enhance transient gene expression in both HEK293 and CHO cell lines^34^ by increasing the overall transcription level while simultaneously inhibiting cell growth^35^. The positive effect of valproic acid (VPA) on transient SEAP expression was confirmed in a time-course study of SEAP expression levels (Fig. 1D). VPA-treated cells analysed by flow cytometry consistently showed a higher level of transfection efficiency and also much higher GFP median fluorescence intensity (data not shown), thus indicating the higher specific productivity of cells transcriptionally activated with VPA. Concurrently, we tried to further boost the expression yield by feeding the cells with casein hydrolysate Tryptone N1 during the production phase, which was previously reported to enhance the productivity of HEK293EBNA cell lines^36^. Peptone additives are often used to replenish nutrients during batch cell culture, and the positive effect of Tryptone N1 (TN1) was also shown in HEK293T cell lines (Fig. 1D). In summary, the optimal values of the transfection and expression parameters were determined for suspension-adapted HEK293T cells through a series of independent experiments, i.e., 1 μg/10^6^ cells for DNA, 1:4 for DNA:PEI ratio, calcium-free DMEM and EX-CELL 293 as transfection and production media, respectively, and the expression yield was further enhanced by adding 2 mM VPA and 0.5% TN1, 3 and 48 hours post-transfection, respectively.

### Construct design and recombinant expression of soluble rat NK cell lectin-like receptors

**Figure 2:**
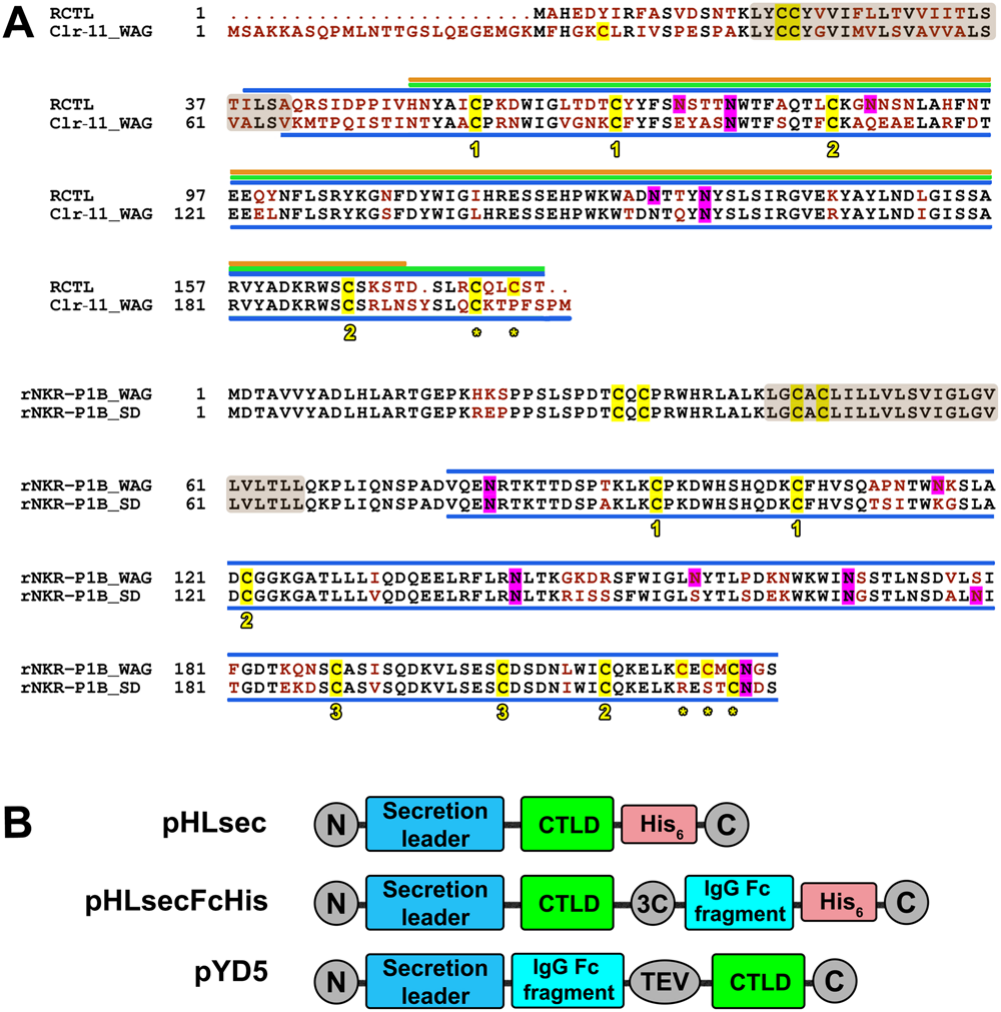
Sequence alignment and design of protein expression constructs. (**A**) Amino acid sequences of rat Clr-11^WAG^, RCTL, NKR-P1B^WAG^, and NKR-P1B^SD^ (GenBank accession nos. DQ168419.1, AF302184, EF100678, and EF100684, respectively) were aligned in ClustalW2; non-identical amino acids are indicated in red; transmembrane regions (as predicted by TMHMM 2.0 server) are highlighted in the brown box; cysteine residues are highlighted in yellow, and cysteine residues conserved among C-type lectin-like receptors and forming intramolecular disulphide bridges are numbered according to their linkage, whereas those expected to form intermolecular disulphide bridges are indicated with an asterisk; potential sites of N-glycosylation are highlighted in magenta; the recombinant protein constructs corresponding to C-type lectin-like domain (CTLD) are indicated with blue lines; the following three constructs were generated in case of RCTL: I38-T180 (blue line), H51-T180 (green line), and H51-T170 (orange line). (**B**) Schematic description of protein expression constructs designed using the three plasmids used in this work.

We selected rat NK cell C-type lectin-like receptors as a model system to study NKR-P1:Clr interactions. In particular, rat inhibitory NKR-P1B receptors from the WAG and SD rat strains differ in several amino acid residues (Fig. 2A), and these differences lead to differential outcomes when WAG or SD rat strains are challenged by cytomegalovirus infection due to different mechanisms of recognition of the viral RCTL decoy ligand^19^. Conversely, Clr-11 ligand from the WAG and SD rat strains differ only in a single amino acid (R192K in the WAG or SD strain, respectively, not shown) located in the C-terminus of the protein and unlikely involved in receptor binding. Therefore, we consider both Clr-11 sequences functionally equal, and we omitted Clr-11^SD^ from the present study. Clr-11^WAG^ and RCTL sequence alignment (Fig. 2A) shows a highly conserved core of the C-type lectin-like domain surrounded by more variable N- and C-terminal sequences of the extracellular portion of the receptor. The C-type lectin-like domain of NK cell receptors usually contains four to eight conserved cysteine residues responsible for intramolecular disulphide bond formation. These residues are necessary to stabilize the domain fold and several additional cysteine residues in the N- or C-terminal chains involved in dimerization by forming an intermolecular disulphide bridge(s). Furthermore, they are presumably required for stable receptor expression at the cell surface. Interestingly, all four receptors contain their proposed dimerization cysteine residues in their C-termini, suggesting that the N-terminal domain chain may not be necessary for dimer formation in these proteins.

**Figure 3:**
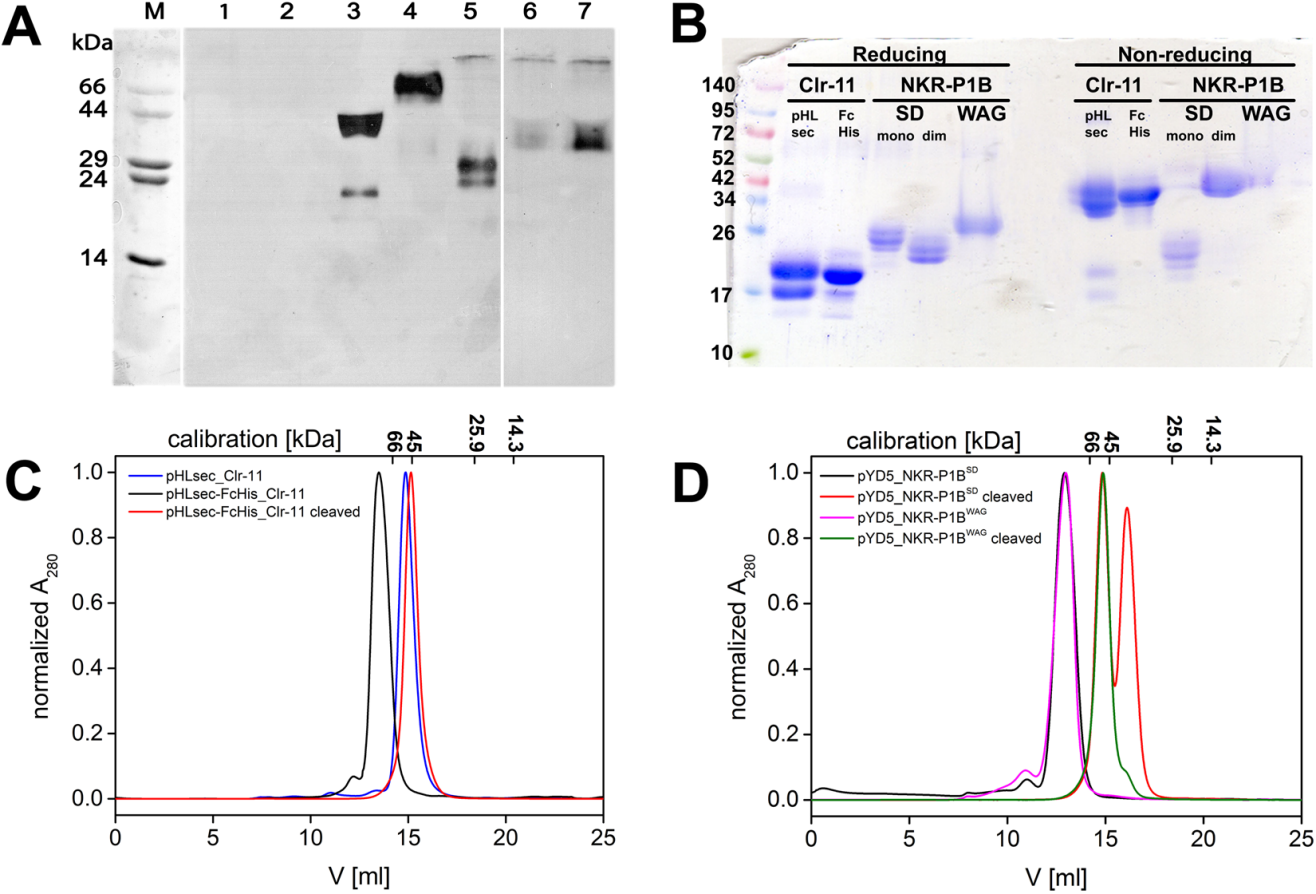
Expression and purification of soluble dimeric rat NK cell C-type lectin-like receptors. (**A**) Small-scale expression test of pHLsec constructs; transfection was performed in a 24-well plate, and 10 μl samples of culture supernatants were collected 3 days later and resolved by 15% SDS-PAGE under non-reducing conditions, transferred onto nitrocellulose membrane and detected by PentaHis mAb; lanes: M, marker; 1, mock transfected; 2, RCTL I38-T180; 3, Clr-11; 4, NKR-P1B^WAG^; 5, NKR-P1B^SD^; 6, RCTL H51-T180; 7, RCTL H51-T170. Full-size images of membranes and their photographs are available as Supplementary Information (Figure S1). (**B**) Samples of purified proteins were resolved by 4–20% SDS-PAGE under both reducing and non-reducing conditions; lanes: pHLsec_Clr-11, pHLsec-FcHis_Clr-11 cleaved off by a 3C protease, pYD5_NKR-P1B^SD^ (monomeric and dimeric fractions) and pYD5_ NKR-P1B^WAG^, both cleaved off by TEV protease. (**C**) Gel filtration profiles of Clr-11, Clr-11-FcHis, and Clr-11 with the FcHis tag cleaved off by a 3C protease. (**D**) Gel filtration profiles of pYD5 Fc-NKR-P1B^SD/WAG^ fusion proteins and NKR-P1B^SD/WAG^ with the Fc tag cleaved off by TEV protease, resulting in the disulphide dimer of NKR-P1B^WAG^ and a mixture of dimer and monomer species of NKR-P1B^SD^.

Initially, we tried to express the whole extracellular part of RCTL, Clr-11^WAG^, and both the NKR-P1B^WAG^ and NKR-P1B^SD^ receptors with only slightly shortened N-termini, i.e., including the stalk region. Thus, four expression constructs were generated by PCR amplification from their respective receptor cDNA templates: RCTL I38-T180, Clr-11^WAG^ V65-M207, NKR-P1B^WAG^ V78-S223, and NKR-P1B^SD^ V78-S223. These constructs were cloned to pHLsec expression vector for secreted protein production (Fig. 2B). The vector adds an additional ETG and GTKHHHHHH amino acid sequences to the N- and C-termini of the construct, respectively^32^. Small-scale expression tests (Fig. 3A, lanes 2–5) showed positive expression for all except for the RCTL constructs, moreover, the NKR-P1B^WAG^ were purely dimeric, Clr-11^WAG^ was mostly dimeric, and NKR-P1B^SD^ was monomeric. To also achieve some expression level for the RCTL protein, two shorter constructs were prepared, RCTL H51-T180 and RCTL H51-T170, and the latter was expressed as a monomer, although at a much lower yield (Fig. 3A, lanes 6 and 7). When expressed by transient transfection in square-shaped bottles and purified by IMAC and gel filtration, the final yield of the pure recombinant receptors ranged from 0.2 to 5 mg per litre of production medium, and Clr-11 and RCTL were the best- and worst-produced proteins, respectively.

### Generation of dimeric Clr-11^WAG^ by C-terminal Fc fusion

Regarding structural biology and protein crystallography, minor heterogeneities, such as incomplete covalent dimerization of Clr-11^WAG^ (Fig. 3B, lanes pHLsec_Clr-11), potentially leaving some flexible parts unstructured, can be detrimental to the quality and usefulness of the final protein preparation. Additionally, glycosylation heterogeneity or the differential occupation of glycosylation sites (the likely reason for the presence of two bands in Clr-11 preparations, Fig. 3B, lanes pHLsec_Clr-11) may increase sample heterogeneity even further. To overcome the first issue, we cloned the Clr-11^WAG^ expression construct into the pHLsec-FcHis vector. In this vector, the C-terminus of the expression construct is fused to human rhinovirus (HRV) 3C protease cleavage site followed by the hinge and the Fc regions of the human IgG1 molecule, and by a hexahistidine tag (Fig. 2B). This FcHis fusion was used to promote the disulphide bond-mediated dimerization of Clr-11 because IgG is a disulphide-linked dimer itself and therefore IgG hinge dimerization may help to position the Clr-11^WAG^ dimerization cysteine residues close enough to enable disulphide bond formation. The expression and purification of Clr-11-FcHis protein was straightforward and at a yield similar to that of the untagged protein, resulting in a purely dimeric fusion Clr-11-FcHis preparation (Fig. 3C, black curve).

The HRV 3C protease preferably cleaves under reducing conditions; however, complete digestion may also be achieved by slightly increasing the protease amount under non-reducing conditions. When applied to the Clr-11-FcHis protein, we observed complete cleavage after one hour at room temperature (data not shown); nevertheless, we chose overnight cleavage at 4 °C for convenience and for lower protease consumption. Because both FcHis fusion and 3C protease contain the histidine tag, we were able to easily separate the cleaved fusion, protease and purely dimeric Clr-11^WAG^ by repeating the purification procedure (Fig. 3C, red curve). Interestingly, the production of Clr-11 with the FcHis fusion also leads to a reduced level of glycosylation heterogeneity, as suggested by the presence of a single band on the SDS-PAGE (Fig. 3B, lane pHLsec-FcHis_Clr-11). When required, glycosylation heterogeneity might be further reduced using N-glycosylation processing inhibitors, such as kifunensine or swainsonine^33^, or the N-acetylglucosaminyltransferase I-negative (GnTI^-^) HEK293S cell line^37^, which is unable to synthesize complex N-glycans.

### Mass spectrometry characterization of Clr-11-FcHis cleavage and dimerization

**Figure 4:**
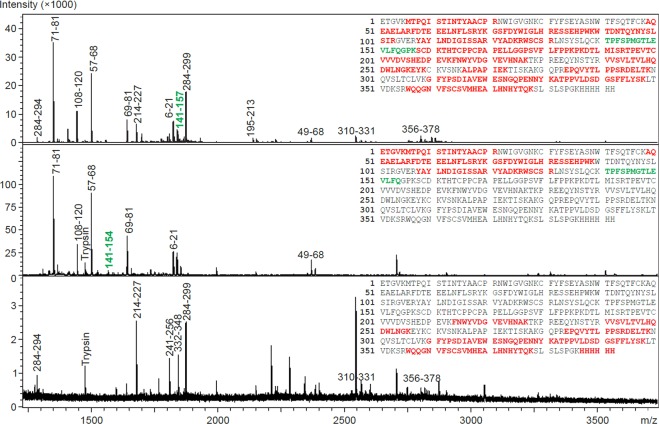
Mass spectrometry analysis of Clr-11-FcHis cleavage with HRV 3C protease. Reduced tryptic digests prepared from SDS-PAGE bands of Clr-11-FcHis (top), Clr-11 with the FcHis fusion cleaved off (middle) and the cleaved FcHis fusion (bottom) were analysed by MALDI-TOF/TOF mass spectrometry. Peptides identified in mass spectra are highlighted in red; peaks in mass spectra are labelled according to corresponding Clr-11-FcHis peptide sequences. The 3C protease cleavage site peptide TPFSPMGTLEVLFQ/GPK is highlighted in green.

**Figure 5:**
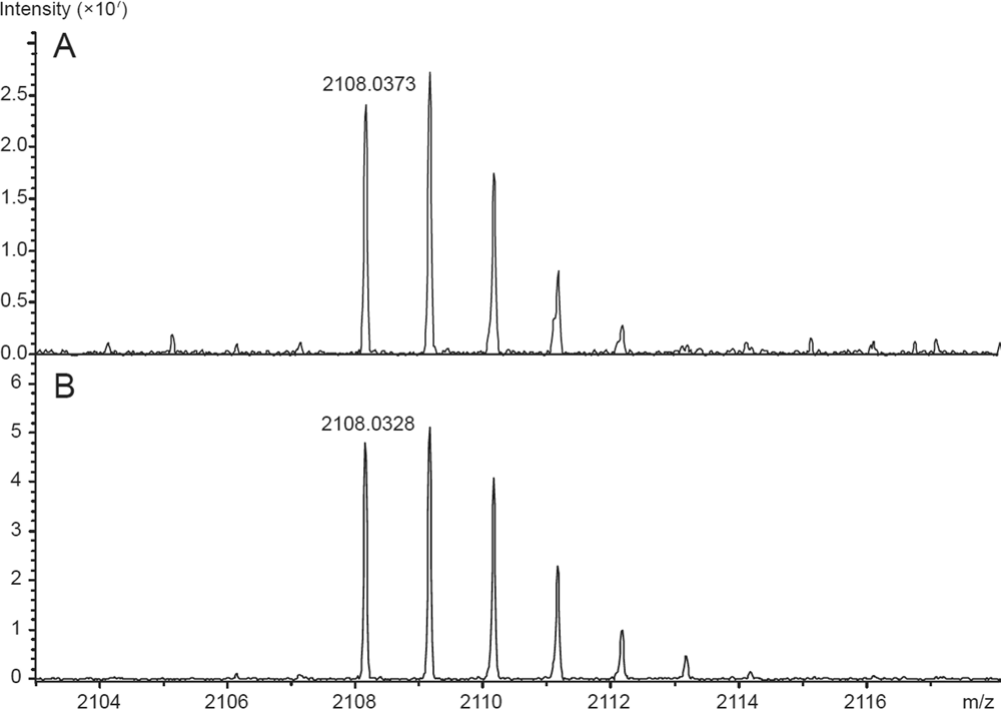
Mass spectrometry analysis of Clr-11 covalent dimerization. MALDI TOF/TOF analyses of tryptic digests of (**A**) Clr-11-FcHis and (**B**) Clr-11 after 3C protease cleavage, both prepared under non-reducing conditions. The m/z value 2108.03 corresponds to the dimerization cystine dipeptide LNSYSLQCK-LNSYSLQCK.

To confirm disulphide bond status and correct covalent dimer formation, we analysed the SDS-PAGE bands of uncleaved and cleaved Clr-11-FcHis proteins by mass spectrometry. The protein bands were excised and digested by trypsin directly within the gel; the resulting peptides were extracted, reduced and analysed in MALDI-TOF/TOF mass spectrometer (Fig. 4). All three samples analysed showed good sequence coverage. The cleavage site TPFSPMGTLEVLFQ/GPK was identified in Clr-11-FcHis but not in Clr-11, where the FcHis fusion was cleaved off and replaced by the TPFSPMGTLEVLFQ peptide, corresponding to the correct fusion cleavage. When examining Clr-11-FcHis and Clr-11 tryptic and Asp-N peptides prepared under non-reducing conditions, we were able to confirm the correct intramolecular disulphide bond connection (data not shown) of all four cysteines present in the C-type lectin-like domain (CTLD), i.e., C80-C91 and C108-C190. More importantly, we were also able to distinguish a cystine dipeptide LNSYSLQCK-LNSYSLQCK, corresponding to the correct covalent intermolecular C200-C200 disulphide bond, and stable dimer formation (Fig. 5).

### Generation of dimeric NKR-P1B by N-terminal Fc fusion

Subsequently, we tried to apply the same approach to NKR-P1B^SD^ and NKR-P1B^WAG^, i.e., to produce these expression constructs as C-terminal FcHis fusions to promote their dimerization. Although we were able to express and purify these fusion proteins in a dimeric form and at a good yield (improved in comparison with the untagged proteins; data not shown), we were unable to efficiently cleave off the FcHis fusion with the 3C protease. A possible explanation is that the dimerization cysteines of the receptor and of the IgG hinge region cross-linked with each other. Thus, although these recombinant fusion proteins are dimeric, their dimerization mode may not be physiological. Reducing the number of cysteine residues in the hinge region or completely removing them from the expression constructs may promote correct dimerization exclusively through the CTLD cysteines.

To overcome this problem, we tested the reverse arrangement of these Fc fusion constructs, i.e., we attached the IgG Fc region to the N-terminus of the NKR-P1B ectodomain using a pYD5 plasmid (Fig. 2B), enabling the subsequent cleavage of the fusion by the tobacco etch virus (TEV) protease. The expression yields of the pYD5_NKR-P1B^SD/WAG^ fusion constructs were similar to those of previous FcHis constructs, and gel filtration showed their correct dimeric state (Fig. 3D, black and magenta curve). Upon cleavage with the TEV protease, we obtained the purely disulphide dimer of the NKR-P1B^WAG^ ectodomain (Fig. 3D, green curve and Fig. 3B, lanes WAG), whereas the NKR-P1B^SD^ ectodomain yielded a mixture of dimeric and monomeric species (Fig. 3D, red curve and Fig. 3B, lanes SD). This difference is likely caused by the fact that NKR-P1B^WAG^ has three cysteine residues in its C-terminus, whereas NKR-P1B^SD^ has a single cysteine in the same position; therefore, disulphide dimerization is more efficient in the NKR-P1B^WAG^ ectodomain.

For the RCTL protein, we were unable to improve its expression yield and dimeric state, even when using FcHis or pYD5 fusion constructs; therefore, we are not yet able to report the successful preparation of any stable soluble form, monomeric or dimeric, of this viral decoy protein in reasonable yield. RCTL is apparently much less stable than its host NK cell counterpart, and its preparation will require further optimization.

### Sedimentation analysis confirms dimeric status of prepared proteins

**Figure 6:**
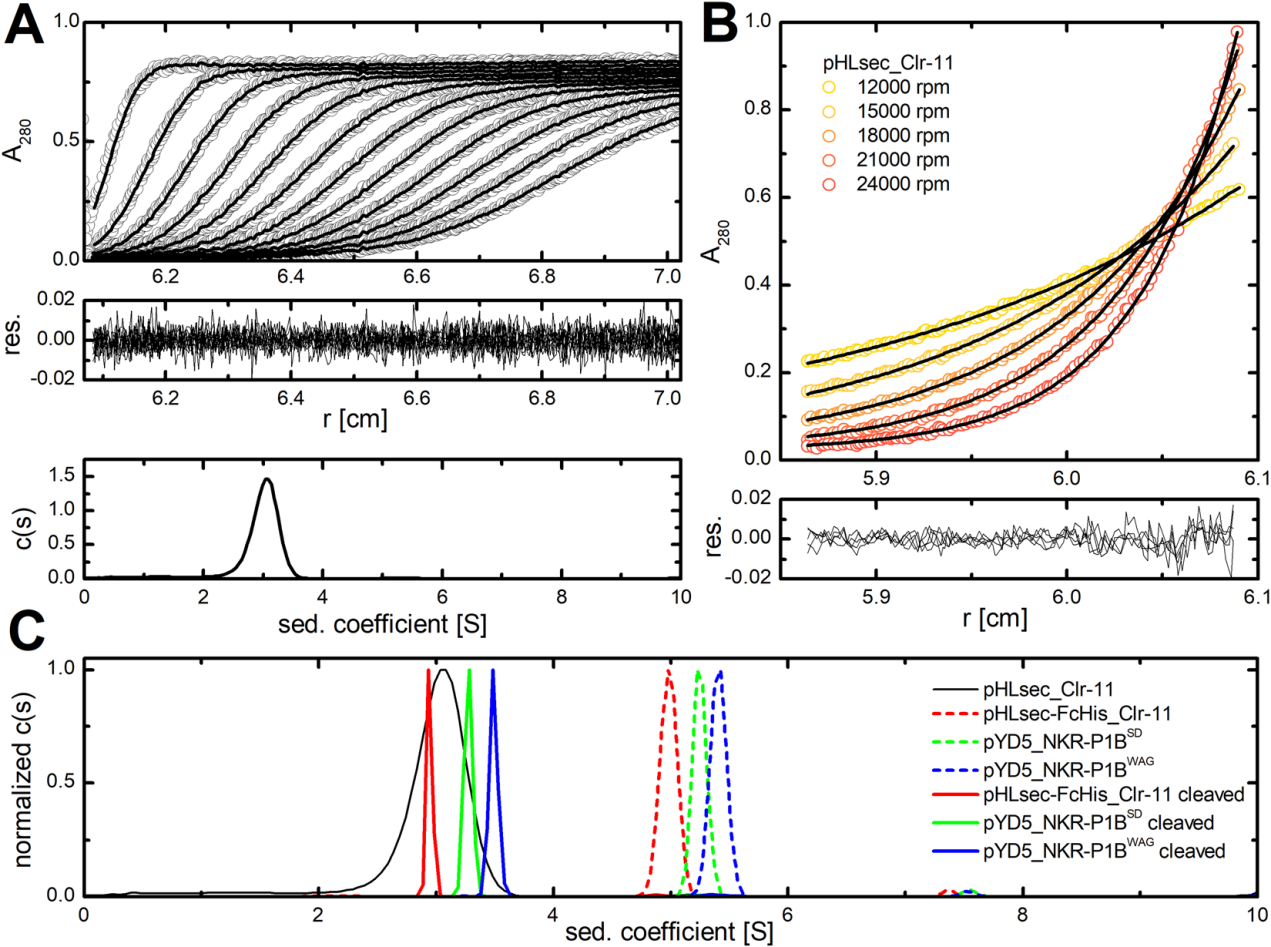
Sedimentation analysis of rat Clr-11 and NKR-P1B dimeric expression constructs. (**A**) pHLsec_Clr-11 protein was analysed by sedimentation velocity; panels show (from top to bottom) absorbance scans recorded at 280 nm (every fifth scan is shown, circles) together with fitted curves (lines), residuals derived from the fitted data, and the resulting c(s) continuous distribution of the sedimentation coefficient. (**B**) pHLsec_Clr-11 was spun at 12–15–18–21–24000 rpm and 4 °C and its sedimentation equilibrium was monitored at 280 nm. The upper panel shows absorbance data (circles) with fitted curves (non-interacting discrete species model, lines), whereas the lower panel shows residuals derived from the fitted data. (**C**) Normalized continuous sedimentation coefficient distributions of pHLsec_Clr-11 (black line), Clr-11 (red line) and both NKR-P1B^SD^ (green line) and NKR-P1B^WAG^ (blue line) cleaved from pHLsec-FcHis and pYD5 Fc fusion constructs, respectively, as well as the distributions of the original uncleaved Fc fusion constructs (the same colour coding, dashed lines).

To further characterize the solution behaviour of the prepared dimeric proteins, sedimentation analysis was performed in an analytical ultracentrifuge. Gel filtration suggested that Clr-11 prepared using a histidine-tagged construct (showing incomplete covalent dimerization, Fig. 3B, lanes pHLsec_Clr-11) and the purely dimeric Clr-11 cleaved from the FcHis fusion construct migrate at the same position (Fig. 3C, blue and red curve), thus suggesting that Clr-11 likely forms a stable non-covalent dimer in solution, even in absence of the stabilizing disulphide bridge. Sedimentation velocity experiments performed with the histidine-tagged Clr-11 showed a single peak in the sedimentation coefficient distribution (Fig. 6A, bottom) with an s_20,w_ value of 3.04 ± 0.2 S, which corresponds to a dimeric protein with a moderately elongated shape and with predicted dimensions of approximately 8–10 × 3–4 nm. However, the broad shape of the peak in sedimentation coefficient distribution suggests the presence of monomer-dimer equilibrium at lower protein concentrations. The weight-average molecular weight of 40837 ± 500 Da was calculated based on the results from the sedimentation equilibrium experiment (Fig. 6B), which also matched the value expected for the glycosylated dimer (2 × 18211 = 36422 Da for the dimeric protein itself + mass of up to 4 N-glycosylation sites occupied per dimer).

Similarly, the sedimentation velocity analyses performed for Clr-11 cleaved from pHLsec-FcHis and for both NKR-P1B^SD^ and NKR-P1B^WAG^ cleaved from pYD5 fusion constructs fully confirmed their entirely dimeric state, yielding s_20,w_ values of 3.10 ± 0.05 S, 3.45 ± 0.05 S and 3.68 ± 0.1 S, respectively (Fig. 6C, solid coloured lines). The shift of these individual sedimentation coefficient values reflects the difference in molecular weight of the individual protein expression constructs and differences in the number of their N-glycosylation sites. Compared with histidine-tagged Clr-11 expressed from the pHLsec vector, Clr-11 cleaved from the pHLsec-FcHis construct is much more homogeneous and shows no sign of monomer-dimer equilibrium (Fig. 6C, black vs. red solid lines). We have also analysed the entire Fc-fusion proteins (Fig. 6C, dashed coloured lines). Their resulting peaks of these ca 100 kDa dimeric fusion glycosylated constructs with apparent sedimentation coefficient values ranging from 5 to 5.5 S correspond to the size and shape of expected elongated particles with estimated dimensions of 13–15 × 4–5 nm. Thus, a fusion of extracellular parts of C-type lectin-like receptors to the Fc region of human IgG is not only an efficient method of preparation of disulphide dimers of these receptors, but also a strategy for the generation of immunoactive constructs with therapeutic potential.

### FTIR spectroscopy analysis of protein secondary structure shows well-folded native proteins

**Figure 7:**
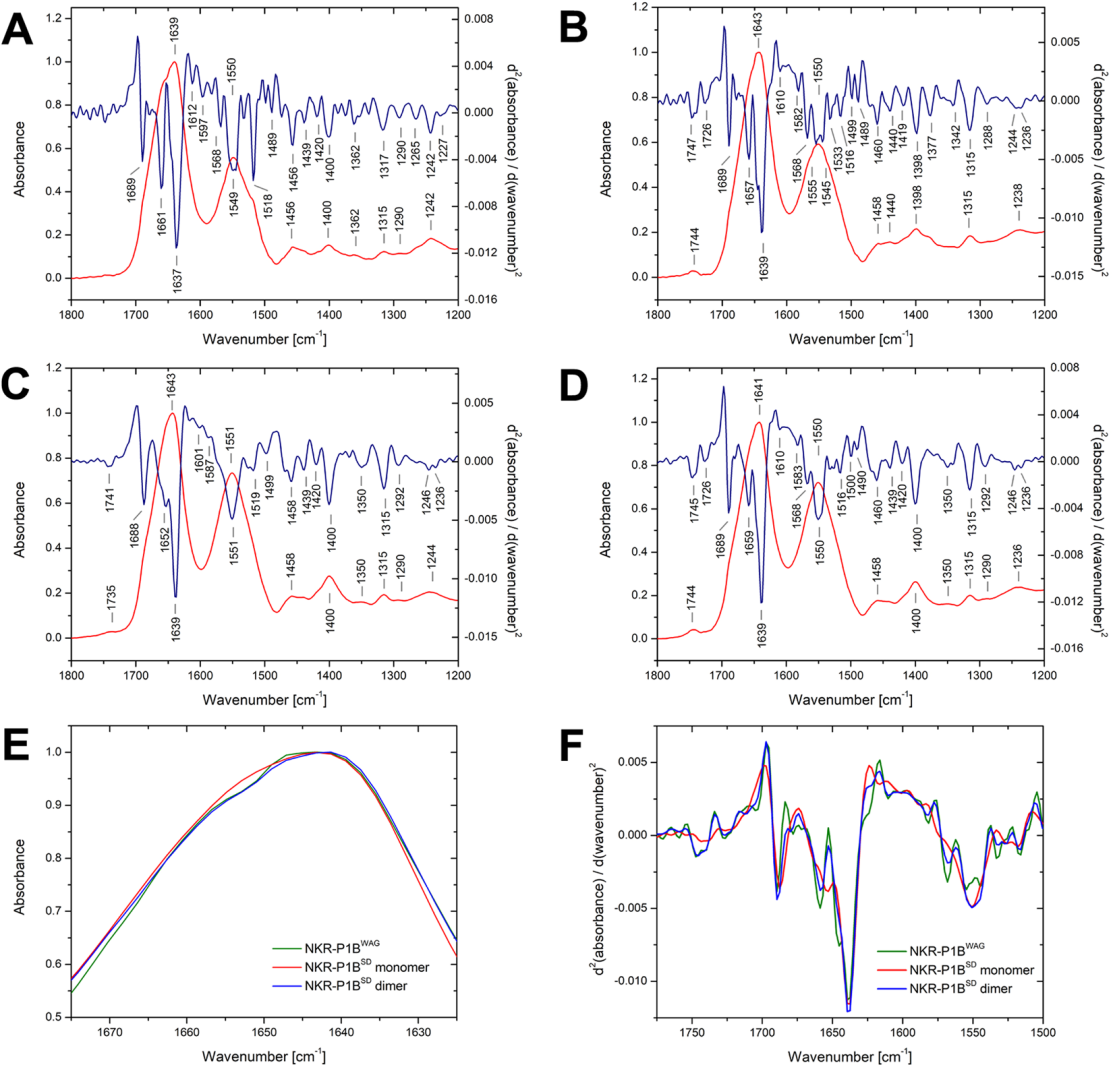
FTIR spectroscopy analysis of recombinant dimeric rat NK cell receptors. Fourier-transform infrared spectra of (**A**) Clr-11 cleaved from pHLsec-FcHis, (**B**) NKR-P1B^WAG^, (**C**) monomeric, and (**D**) dimeric NKR-P1B^SD^, all cleaved from pYD5 fusion constructs, in the region of amide I, II and III bands depicted as red curves. The blue line corresponds to the second derivative of the spectrum smoothed by Savitski-Golay function at 7 points; distinct spectral bands are labelled, and band assignment is outlined in Table 1. (**E**) Detailed comparison of the FTIR spectra and their (**F**) second derivatives for monomeric and dimeric NKR-P1B^SD^, and NKR-P1B^WAG^ in the region of amide I and II bands.

Lastly, the secondary structure of all recombinant soluble receptor constructs was examined by Fourier-transform infrared (FTIR) spectroscopy (Fig. 7 and Table 1). The FTIR spectra of all studied proteins are dominated by two broad bands corresponding to vibrations of the peptide linkage – amide I at ~1639 cm^−1^ (dominated by νCO, i.e., stretching vibrations of CO group) and amide II at ~1550 cm^−1^ (dominated by δNH, i.e., bending vibrations of the NH group, and νCN; Fig. 7A–D). The overall character of these spectra and the maximum of the amide I band correspond to proteins dominated by β-sheet structure. The intensity ratio of amide I and amide II bands, which reflects presence of aggregates, corresponds to fully soluble globular proteins, and no marks of aggregated structures are present (amide I/amide II ratio is close to 3:2 in soluble globular proteins and 1:1 in aggregated structures, whereas the band of intermolecular/aggregated β-sheets at ~1620 cm^−1^ is missing). The second derivative of Clr-11 FTIR spectra clearly shows a high content of β-sheets by the strong negative band at 1637 cm^−1^, thus confirming that Clr-11 is structurally similar to mouse Clr-g.

**Table 1:** Assignment of the infrared bands distinguishable in the second derivatives of Clr-11/monomeric NKR-P1B^SD^/dimeric NKR-P1B^SD^/NKR-P1B^WAG^ receptors FTIR spectra shown in Fig. 7 (ν corresponds to stretching, δ to bending, *as* to anti-symmetrical, and *s* to symmetrical vibrations).

Frequency (cm^−1^)	Assignment	References
1227/—/—/—	β-sheet (amide III)/His	^39,54–56^
1242/1236/1236/1236	β-sheet (amide III)/Tyr–OH	^39,54–56^
1265/1246/1246/1244	coil (amide III)/Tyr–OH	^39,54–56^
1290/1292/1292/1288	β-turns (amide III)	^54,55^
1317/1315/1315/1315	α-helix (amide III)	^54,55^
1362/1350/1350/1342	Trp	^39,56^
1400/1400/1400/1398	Asp/Glu (ν_s_COO^–^)	^39,56^
—/—/—/1419	Trp/Pro?	^39,56^
1439/1439/1439/1440	δCH_2_, Pro (νCN), His^–^(δCH & δCN)	^39,56^
1456/1458/1460/1460	δCH_2_, δCH_3_/Tyr/Trp?	^39,56^
1489/—/1490/1489	Trp? (νCC ring & δCH)	^39,56^
—/1499/1500/1499	Phe/Tyr–O^–^ (νCC ring)?	^39,56^
1518/1519/1516/1516	Tyr–OH (νCC ring & δCH)	^39,56^
—/—/—/1533	amide II	^40,56^
—/—/—/1545	amide II	^40,56^
1549/1551/1550/—	amide II	^40,56^
—/—/—/1555	amide II	^40,56^
1568/—/1568/1568	Asp/Glu (ν_as_COO^−^)	^39,56^
1597/1587/1583/1582	Tyr (νCC ring)	^39,56^
—/1601/—/—	Tyr–O^–^ (νCC ring)?	^39,56^
—/—/1610/1610	Tyr (νCC ring)	^39,56^
1637/1639/1639/1639	β-sheet (amide I)	^40,57^
1661/1652/1659/1657	α-helix/coil/turns (amide I)	^40,57^
1689/1688/1689/1689	β-sheet antiparallel/ turns (amide I)	^40,57^
—/—/1726/1726	Asp/Glu (νC=O)	^39,54,56^
—/1741/1745/1747	Asp/Glu (νC=O)	^39,54,56^

The results of secondary structure analysis are summarized in Table 2. The differences between the original FTIR spectra and the fitted curves taken from the protein spectra reference set are very low (lower than 6%, not shown). This translates into reasonable estimations of the protein secondary structure because the relative sums of the estimated structures are < 110%. All estimated secondary structures are very close to each other, as expected for proteins belonging to the same structural family. The estimated secondary structures of monomeric and dimeric NKR-P1B^SD^ are identical, and also the NKR-P1B^WAG^ structure differs only very little. The secondary structure estimated for dimeric NKR-P1B^SD^ in solution and calculated from its crystal structure is very close and inside the margins of errors, which is also true for soluble rat Clr-11 and for the crystal structure of the closely related mouse Clr-g. The values estimated in this study for the dimeric NKR-P1B^SD^ are also very close to values previously estimated for a monomeric, bacterially expressed and refolded, shorter expression construct of rat NKR-P1B^38^.

**Table 2:** Estimation of the secondary structure content of the prepared proteins using the least-squares method (LSA)^51^ analysing amide I & II bands in infrared spectra (*N* marks values normalized to 100%).

NKR-P1B^WAG^		Clr-11		mouse Clr-g	
Structure	LSA (%)	LSA *N* (%)	LSA (%)	LSA *N* (%)	crystal structure*
α-helix	21 ± 10	20	23 ± 10	22	17
β-sheet	28 ± 9	27	25 ± 9	24	27
β-turn	14 ± 4	13	13 ± 4	12	12
Bend	15 ± 4	14	14 ± 4	13	15
Disordered	27 ± 6	26	30 ± 6	29	29
Sum	105%	100%	105%	100%	100%
**NKR-P1B**^**SD**^ **monomer**		**NKR-P1B**^**SD**^ **dimer**		**NKR-P1B**^**SD**^ **dimer**	
**Structure**	**LSA (%)**	**LSA** ***N*** **(%)**	**LSA (%)**	**LSA** ***N*** **(%)**	crystal structure*
α-helix	23 ± 10	22	23 ± 10	22	19
β-sheet	28 ± 9	26	28 ± 9	26	28
β-turn	14 ± 4	13	14 ± 4	13	15
Bend	16 ± 4	15	16 ± 4	15	13
Disordered	26 ± 6	24	26 ± 6	24	25
Sum	107%	100%	107%	100%	100%

Given standard deviations are calculated as standard deviations of the used reference set; therefore, they do not reflect the quality of the fits. *Calculated using DSSP^58^ on the crystal structures of mouse Clr-g (PDB ID 3RS1)^29^ and rat NKR-P1B^SD^ dimer (PDB ID 5J2S). However, in the NKR-P1B^SD^ crystal structure, only 118 amino acid residues are visible, i.e., 29 residues (20%), fewer than those present in the expressed protein construct.Given standard deviations are calculated as standard deviations of the used reference set; therefore, they do not reflect the quality of the fits.

Although the NKR-P1B^SD^ and NKR-P1B^WAG^ dimers are highly similar – both show the same pattern in amide I and II regions – a slight, yet distinct, difference between the NKR-P1B^SD^ monomer and the corresponding dimer is visible at ~1655 cm^−1^ (Fig. 7E), even though their secondary structures show no significant differences (Table 2). The differences are best observed when using the second derivatives (Fig. 7F). The band at 1652 cm^−1^ is shifted at 1659 cm^−1^ upon NKR-P1B^SD^ dimer formation. Unfortunately, this band shift might be attributed both to α-helices or disordered structures^39^, or even partly to turns^40^. Slight changes in turns are observed in the region at ~1689 cm^−1^. However, no changes in β-sheet bands are identified. Thus, β-sheets unlikely participate in NKR-P1B^SD^ dimerization, further confirming that the NKR-P1B^SD^ dimer structure in solution is the same as the crystal structure in which the dimerization interface is formed by α-helices and surrounding turns and loops without involving the β-sheet protein core.

## Discussion

Although new detailed structural and functional data are revealing the functional role of NKR-P1 monomers and dimers and the nature of the NKR-P1:Clr interaction, difficulties producing stable covalent NKR-P1 homodimers require using recombinant NKR-P1 proteins from different host organisms in a different stoichiometry. Recently, Hernychová *et al*. reported that only monomeric forms of mouse NKR-P1B could bind to Clr-b on cell surface, suggesting that NKR-P1B monomers might separately participate in the interaction with Clr-b or that the interaction itself might promote NKR-P1B dimerization^41^. Although the signalling efficiency of monomeric NKR-P1B was not evaluated in this study, Hernychová *et al*. concluded that NKR-P1B homodimers are not functional, regardless of the presence of a stalk region, which did not affect the interaction and was not even crucial for protein dimerization. Remarkably, in another recent publication Balaji *et al*. showed that only homodimers of mouse NKR-P1B were able to functionally engage CD3ζ-Clr-b chimeras on cells^42^. Moreover, in contrast to the Hernychová *et al*. findings, Balaji *et al*. reported that abrogation of the intermolecular cystine bond was detrimental to functional signalling, precluding the NKR-P1B self-association and higher-order cross-linking of Clr-b. However, NKR-P1B homodimers were not essential for the formation of a stable NKR-P1B:m12 viral immunoevasin complex, most likely because the NKR-P1B:m12 complex has a much higher affinity than the NKR-P1B:Clr-b complex. Unfortunately, discrepancies in the preparations of mouse NKR-P1B protein prevent direct comparisons between these results. Soluble, renatured NKR-P1B, produced in *E*. *coli*, which results in a mixture of monomers and multiple species of covalent homodimers, was used in the first study^41^, whereas Balaji *et al*. was unable to produce covalent homodimers of NKR-P1B in HEK293 cells and instead used tetrameric NKR-P1 particles consisting of four streptavidin-bound biotinylated NKR-P1B monomers.

To meet this need, we developed a rapid method for the preparation of stable soluble covalent NKR-P1 dimers in HEK293 cells suitable for further functional studies and offering therapeutic potential thanks to the presence of IgG Fc fragment, if desired. First, we adapted HEK293T cells for growth in suspension in EX-CELL 293 medium supporting high-density cultures. When compared with adherent cultures, suspension cultures have the potential to be more economical (and ecological) due to their lower requirements for single-use plastic consumables and higher volumetric productivity. However, the likely presence of negatively charged additives (e.g., heparin) may have contributed to ineffective PEI-mediated transfection of HEK293T cells in EX-CELL 293 medium. This is however not a problem in F17 or calcium-free DMEM media in which we were able to optimize transfection parameters (i.e., 1 μg plasmid DNA/10^6^ cells, 1:4 (w/w) DNA:PEI ratio, and calcium-free DMEM and EX-CELL 293 as transfection and production media, respectively), and to confirm the positive effect of 2 mM valproic acid added 3 hours post-transfection, as well as of Tryptone N1 added 48 hours post-transfection, on the yield of secreted protein.

To demonstrate the feasibility of preparing covalent disulfidic NKR-P1B dimers in our optimized expression system, we selected the rat inhibitory NKR-P1B:Clr-11 as a model receptor:ligand system – particularly because NKR-P1B receptors from WAG and SD rat strains share their native ligand Clr-11 but differ in their reactivity towards the viral decoy ligand RCTL^19^. Although we have been able to produce dimeric NKR-P1B^WAG^ and mostly dimeric Clr-11, we were unable to obtain covalent dimers of the NKR-P1B^SD^ and RCTL molecules. The RCTL viral decoy ligand proved especially difficult to express, and we obtained only low yields of its monomeric form. The varied dimerization propensity of the NKR-P1B^WAG^ and NKR-P1B^SD^ receptor ectodomains is proportional to the number of available C-terminal dimerization cysteines – the NKR-P1B^WAG^ has three such cysteine residues, whereas NKR-P1B^SD^ contains only one.

Nevertheless, to further promote the covalent dimerization of soluble Clr-11 and NKR-P1B^SD^, we have prepared fusion constructs with a C-terminally attached Fc fragment of the human IgG1 molecule, cleavable with HRV 3C protease. The C-terminal fusion of Fc fragment was a successful strategy in the case of Clr-11, resulting in stable Cys200-Cys200 bound Clr-11 covalent dimer, as confirmed by mass spectrometry. However, we were unable to cleave off the C-terminal Fc fragment from both NKR-P1B isoforms, most likely due to cross-linking of IgG cysteines with the C-terminal dimerization cysteines of the receptor. This problem caused by the proximity of the C-terminal NKR-P1B to the Fc IgG cysteines was solved by fusing the Fc fragment to the N-terminus of the NKR-P1B ectodomain, thereby efficiently cleaving off the N-terminal Fc fragment and yielding pure NKR-P1B^WAG^ covalent dimer and a separable mixture of NKR-P1B^SD^ monomer and covalent dimer. Thus, we do not recommend C-terminal Fc fragment fusion for rat NKR-P1 expression because it can result in the formation of non-physiological dimers. Conversely, N-terminal Fc fragment fusion is a suitable expression strategy to quickly prepare covalent rat NKR-P1 dimers in milligram quantities.

Further gel filtration and sedimentation velocity analyses showed that monomeric Clr-11 exhibits an equilibrium of monomers and non-covalent dimers in solution, as expected because several *Clec2* orthologues, including mouse Clr-g^29^ and Clr-b^42^ and human LLT1^43^ and CD69^44^, have been shown to form non-covalent dimers in solution. Furthermore, the prepared covalent dimers of NKR-P1B^WAG^, NKR-P1B^SD^ and Clr-11 showed no sign of monomer-dimer equilibrium, thus corroborating the efficiency of our expression strategy. The prepared receptors were also examined by FTIR spectroscopy clearly showing fully soluble globular proteins with no signs of aggregation. Furthermore, analysis of FTIR spectra confirmed the structural similarity of rat Clr-11 to mouse Clr-g. However, the comparison of FTIR spectra of monomeric and dimeric NKR-P1B^SD^ showed that its β-sheets are unlikely to participate in NKR-P1B^SD^ dimerization. This finding corroborates the crystal structure of NKR-P1B^SD^ (PDB ID 5J2S – Vaněk *et al*., manuscript in preparation), wherein the dimerization interface is mainly formed by α-helices. Combined with the recently reported unconventional dimerization modes of mouse NKR-P1B^41,42^, these results indicate that dimerization within the NKR-P1 receptor family differs from that observed in *Clec2* ligands.

Several factors likely hinder the effective formation of covalent dimers of soluble NKR-P1 constructs. When compared with the Clr receptor family, the weaker mode of non-covalent dimerization of NKR-P1B, as revealed in its crystal structures (Balaji *et al*.^42^ and PDB ID 5J2S), has rather weak affinity. Therefore, membrane anchoring of the NKR-P1B molecule may be required for the effective formation of disulphide bonds within its stalk region. Thus, by providing such steric anchoring through N-terminal fusion to the Fc-fragment, the dimerization cysteines can be brought into functional proximity and promote the formation of disulphide bridges. The low propensity for non-covalent dimerization is apparently a common feature of NKR-P1 molecules and suggests that the weaker non-conventional mode of dimerization might be conserved throughout the NKR-P1 family. This overall conformational flexibility of NKR-P1 molecules might be also the reason why it is practically impossible to detect and measure the binding of rodent NKR-P1 receptors to their respective Clr ligands in solution. Apparently, the affinity of individual NKR-P1:Clr interaction is extremely weak and largely undetectable by standard biophysical approaches^42^. Yet such behaviour is not uncommon among immune receptor:ligand complexes where it is the interaction avidity (provided by, e.g., cross-linking of the dimeric receptor:ligand molecules on the cell surface) that plays a major role in signal transduction to the cell.

In summary, the method described herein enables high-level expression of secreted recombinant soluble dimeric forms of rat NK cell C-type lectin-like receptors in quantity and quality sufficient for their biophysical, functional, and structural characterization. Transient transfection is an easily scalable, non-viral, fast and affordable method of recombinant protein production in HEK293 cell lines that allows us to use modular construct design. Furthermore, the fusion of receptor expression constructs to an Fc fragment of human IgG promotes receptor disulphide dimer formation and could be used for purification, detection, or therapy. This approach can be applied to generate other soluble NK cell surface antigens, thus enabling their detailed structural and functional characterization leading to detailed molecular insights necessary for successful rational design of new protein-based immunotherapeutics.

## Methods

### Chemicals

25-kDa linear polyethylenimine (Polysciences, USA) was dissolved in water, neutralized with HCl, sterilized by filtration (0.22 μm), aliquoted and stored at −80 °C; a working aliquot was stored at 4 °C. Valproic acid and Pluronic F-68 (both Sigma, USA) were dissolved in water to 0.5 M and 10% (w/v), respectively, sterilized by filtration and stored at −20 °C. Casein hydrolysate Tryptone N1 (Organotechnie, France) was dissolved in F17 medium (GIBCO Invitrogen, USA) to 20% (w/v), sterilized by filtration and stored at 4 °C.

### Cell culture

HEK293T cells were kindly provided by Radu A. Aricescu^32^ and were maintained as adherent monolayers in standard Dulbecco’s Modified Eagle’s Medium (DMEM, 4.5 g/l glucose, Institute of Molecular Genetics, The Czech Academy of Sciences, Prague) supplemented with 4 mM L-glutamine, non-essential amino acids and 10% foetal bovine serum (GIBCO Invitrogen, USA) in standard flasks (TPP, Switzerland) in a humidified 37 °C, 5% CO_2_ incubator. Suspension adapted HEK293T cells (0.25–6 × 10^6^/ml) were maintained in EX-CELL 293 serum-free medium (Sigma, USA) supplemented with 4 mM L-glutamine in standard dishes (TPP, Switzerland) or square-shaped glass bottles with gas permeable caps (DURAN, Germany) using 30–40% of the nominal volume at 135 rpm (Orbit 1000 orbital shaker, rotational diameter 19 mm; Labnet, USA; bottles were fixed with Sticky Pad adhesive mat; New Brunswick Scientific, USA) placed within the same incubator^45^.

### Vectors, cloning, and DNA purification

The pTTo3c-SSH and pTTo-GFPq vectors containing secreted alkaline phosphatase and green fluorescent protein, respectively, were kindly provided by Dr. Yves Durocher, as well as pYD5 vector (pTT5 derivative with N-terminal human IgG Fc fragment tag cleavable by TEV protease that was in-house modified to contain AgeI/KpnI cloning sites)^31^. The pHLsec and pHLsec-FcHis vectors were kindly provided by Dr. Radu A. Aricescu^32^. Isolation of cDNA of rat NKR-P1B, Clr-11, and RCTL receptors was previously described^19^. Briefly, PCR products were digested, purified and inserted into pHLsec, pHLsec-FcHis or pYD5 vectors using the flanking AgeI and KpnI sites. Positive clones were screened by colony PCR using TaqRed PCR master mix (Top-Bio, Czech Republic). The primers used to amplify the desired expression constructs and the vector-specific primers are listed in Supplementary Information (Table S1). All inserts were sequenced, and the plasmid transfection stocks were prepared using NoEndo JETSTAR 2.0 Plasmid Maxiprep Kit (Genomed, Germany) according to the manufacturer’s recommendations. Using a single kit column, 3–4 mg of pure plasmid DNA (A_260_/A_280_ ratio of 1.8–2.0) was usually obtained from 500 ml of *E*. *coli* DH5α culture grown in Luria broth medium (in our experience, cultures grown for more than 12-14 h give lower yields).

### Small-scale transient transfections

Cells were centrifuged and resuspended in appropriate fresh transfection medium at a density of 0.5 × 10^6^/ml. Transfection medium was calcium-free DMEM (as above, but calcium chloride and FBS were not used in the preparation; instead, 0.1% Pluronic F-68 was added) either supplemented with 2% FBS or completed with EX-CELL 293 post-transfection (see below). Alternatively, F17 (supplemented with 4 mM L-glutamine and 0.1% Pluronic F-68) or Glutamax-I Freestyle 293 medium (both GIBCO Invitrogen, USA) were used, as indicated in Results, and 0.5 ml of cell suspension was distributed per well in a 24-well plate (or 1 × 10^6^/ml in case of calcium-free DMEM and 0.25 or 1 ml of cell suspension was distributed per well in a 24- or 12-well plate, respectively). The desired amount of DNA (1 μg/10^6^ cells unless otherwise noted) was diluted in PBS (in a volume equivalent to one-tenth of the culture to be transfected), PEI was added to desired ratio (w/w; 1:4 unless noted otherwise), and the mixture was immediately vigorously shaken and incubated for 10–15 min at room temperature before adding it to the cells. Following a 3 h incubation with DNA-PEI complexes, the culture medium was completed to 0.5 ml (24-well plate) or 2 ml (12-well plate) with EX-CELL 293 in case of calcium-free DMEM transfections, and VPA was added (concentrations indicated in the text refer to a final culture volume).

### SEAP analysis

SEAP activity (∆A_410_/min) was determined as previously described^31^. Briefly, culture supernatants were diluted with water as required (typically 1/100 to 1/1000), and 180 μl was transferred to a 96-well plate. The enzymatic reaction was initiated when 20 μl of SEAP assay solution (20 mM *p*-nitrophenyl phosphate; pNPP, 1 mM MgCl_2_ and 1 M diethanolamine pH 9.8) were added, and absorbance was read at 410 nm in 1 min intervals at room temperature to determine the pNPP hydrolysis rates (Safire microplate reader, Tecan, Austria). Data are expressed as the mean of one experiment performed in triplicate with error bars representing standard deviations. Each sample was independently assayed for SEAP activity three times to minimize pipetting errors.

### Flow cytometry

GFP-positive viable cells were estimated using a BD LSR II flow cytometer (BD Biosciences, USA). For each assay, 50 μl of cell suspension were transferred to a round-bottom 96-well plate, diluted with 150 μl of PBS and stained with 10 μl of propidium iodide (PI; 10 μg/ml in PBS) before analysis. Viable transfected cells were quantified using appropriate gating to exclude dead cells, debris and aggregates in a forward vs. side scatter plot. Data are shown as mean of one experiment performed in triplicate with error bars representing standard deviations.

### Transfection in square-shaped bottles

For large-scale recombinant protein production, the respective expression plasmid (1 μg/10^6^ of cells to be transfected, typically 400 μg) was diluted in 10 ml of PBS, filter sterilized, and PEI was added at a 1:4 weight ratio (typically 1.6 mg). The mixture was then shaken and incubated for 10 min. Meanwhile, exponentially growing HEK293T cells were centrifuged and resuspended in calcium-free DMEM at a density of 2 × 10^6^/ml (typically in 200 ml of medium in a 1 l square-shaped bottle) and immediately transfected. Following a 3–4 h incubation period with DNA-PEI complexes, the culture medium was completed with an equal volume of EX-CELL 293 (typically 200 ml), and VPA was added to 2 mM concentration. Two days later, the culture was fed with 0.5% TN1. Conditioned culture medium containing secreted recombinant protein was harvested by centrifugation 4–6 days post-transfection and stored at −20 °C until protein purification.

### Protein purification

Conditioned medium was thawed, clarified by centrifugation at 25000 × g, and filtered through a 0.22 μm membrane (Steritop filter, Millipore, USA). The medium was diluted twofold with PBS, and the final pH was adjusted to 7.0, when necessary. IMAC purification was performed using cobalt-coated TALON beads (Clontech, USA) in batch mode using 2 l Erlenmeyer flasks. Following a 30-min incubation period with shaking at 110 rpm, the beads were collected in a gravity flow Econo column (Bio-Rad, USA), washed with PBS and the bound His-tagged protein was eluted with PBS containing 250 mM imidazole. The eluate was concentrated with Amicon Ultra device (10 kDa cut-off membrane, Millipore) and subjected to gel filtration on Superdex 200 HR 10/30 column (GE Healthcare, USA). All steps were performed at room temperature. Protein concentration was determined by Bradford assay (Bio-Rad). All pHLsec-FcHis constructs were cleaved with HRV 3C protease overnight, at 4 °C, in non-reducing conditions at a 1:5 target-to-protease mass ratio; all pYD5 constructs were captured on a Protein A column (MabSelect SuRe, GE Healthcare, USA), followed by cleavage with TEV protease and purification by gel filtration, as described above.

### Electrophoresis and Western blot analysis

For the rapid screening of new constructs, transfections were performed in 24-well plates using miniprep-purified DNA (JETQUICK Spin Kit, Genomed). Three days post-transfection, 10 μl of conditioned media was resolved on a 15% SDS-PAGE gel, which was subsequently electroblotted onto a BioTrace nitrocellulose membrane (Pall Corporation, USA), followed by Ponceau Red staining and washing with TBS buffer (10 mM Tris, pH 7.5, 150 mM NaCl). The membrane was blocked with 3% BSA in TBS for 1 h at room temperature, and thrice rinsed with TBS-T buffer (20 mM Tris, pH 7.5, 500 mM NaCl, 0.05% Tween-20, 0.2% Triton X-100). After 1 h incubation with PentaHis monoclonal primary antibody (1:1000 dilution in 3% BSA in TBS, Qiagen, Germany) and another 1 h incubation with horseradish peroxidase-conjugated goat anti-mouse IgG polyclonal antibodies (1:2000 in 10% non-fat milk in TBS; Abcam, UK), with extensive washings with the TBS-T and TBS buffers after each step, peroxidase activity was visualized by luminol chemiluminescence.

### Mass spectrometry analysis

Mass spectrometry analysis of disulphide bond linkage was performed as previously described^46^. Briefly, to avoid disulphide bond scrambling, 0.2 mM cystamine was added at all stages of sample preparation (i.e., SDS-PAGE gels, sample, running and digestion buffers). Protein bands were excised from the gel and their tryptic and Asp-N peptide digests were extracted and analysed on a MALDI-TOF/TOF mass spectrometer (ULTRAFLEX III, Bruker, Germany). Experimentally determined *m*/*z* values were compared with theoretical values created in GPMAW software^47^, and cystine peptides were identified using a software LinX (freely available at http://peterslab.org/MSTools/). Routine protein identification and HRV 3C or TEV protease cleavage analysis was similarly performed with reduced tryptic digests.

### Sedimentation analysis

The native molecular size and shape as well as the molar mass of the proteins produced were analysed in an analytical ultracentrifuge ProteomeLab XL-I (Beckman Coulter, USA) using both sedimentation velocity and sedimentation equilibrium experiments. Before the experiment, protein samples were diluted with the gel filtration buffer (10 mM HEPES, pH 7.0, 150 mM NaCl, 10 mM NaN_3_) to 0.2 mg/ml concentration, and the buffer was used as a reference. Sedimentation velocity experiments were conducted at 48000 rpm and at 20 °C using double-sector cells and An50-Ti rotor. Absorbance scans were recorded at 280 nm, at 5-min intervals. Buffer density, protein partial specific volume, and particle dimensions were estimated in Sednterp (www.jphilo.mailway.com). Data were analysed in Sedfit^48^ using a continuous sedimentation coefficient distribution c(s) model. The sedimentation equilibrium experiment was performed with pHLsec_Clr-11 at a concentration of 0.1 mg/ml at 12–15–18–21–24000 rpm and at 4 °C in a six-sector cell, and absorbance scans were collected after 36 h (first scan) or 20 h (consecutive scans) of equilibration. The sedimentation equilibrium data were globally analysed in Sedphat^49^ using a non-interacting discrete species model.

### Fourier-transform infrared spectroscopy

The proteins were transferred to 10 mM Tris, pH 7.5, 50 mM NaCl buffer using centrifugal filters (10 kDa cut-off, 12000 × g, Millipore, USA) at 20 mg/ml final concentration. Infrared spectra were recorded within a Vector 33 FTIR spectrometer (Bruker, Germany) using a standard MIR source, KBr beam-splitter and a DTGS detector. For each sample 4000 scans were collected with a 4 cm^−1^ spectral resolution using a Blackman-Harris 3-term apodization function. Protein samples were measured at room temperature in a CaF_2_-cell with an 8-µm path length (Chevtchenko Optics, Germany). The spectrometer was purged by dry air during all experiments. The spectral contribution of the buffer was corrected following the standard algorithm^50^. The spectrum of water vapour was subtracted; the spectra were offset at 1800 cm^−1^ at zero and normalized to the amide I intensity maximum at one. Data were processed using the software GRAMS/AI (Thermo Electron, USA). The secondary structure of the proteins was estimated from their infrared spectra using the Dousseau and Pézolet method^51^ implemented as a Matlab routine (MathWorks, USA) in the Vibrational Spectroscopy Toolbox and Applications^52^. This method uses least-squares analysis to compare the amide I and amide II bands of a protein of unknown structure with those of the reference set of proteins of known three-dimensional structure (taken from^53^). The main advantage of this method is its independence from band assignments.

## Supplementary information

Supplementary Information

## Data Availability

All data generated or analysed during this study are included in this published article. Raw data, e.g., mass and FTIR spectra or AUC datasets generated in the current study are available from the corresponding author on reasonable request.
