# Publisher Correction: Production of recombinant soluble dimeric C-type lectin-like receptors of rat natural killer cells

**DOI:** 10.1038/s41598-020-59778-7

**Published:** 2020-02-13

**Authors:** Ondřej Vaněk, Petra Celadova, Ondřej Skořepa, Jan Bláha, Barbora Kalousková, Anna Dvorská, Edita Poláchová, Helena Pucholtová, Daniel Kavan, Petr Pompach, Kateřina Hofbauerová, Vladimír Kopecký, Aruz Mesci, Sebastian Voigt, James R. Carlyle

**Affiliations:** 1 Department of Biochemistry, Faculty of Science, Charles University, Hlavova 2030/8, 12840 Prague, Czech Republic; 2 Institute of Microbiology, The Czech Academy of Sciences, Vídeňská 1083, 14220 Prague, Czech Republic; 3 Institute of Physics, Faculty of Mathematics and Physics, Charles University, Ke Karlovu 5, 12116 Prague, Czech Republic; 4 Department of Immunology, University of Toronto, 1 King’s College Circle, M5S 1A8 Toronto, ON Canada; 5 Department of Infectious Diseases, Robert Koch Institute, Seestraße 10, 13353 Berlin, Germany; 6 Present Address: EMBL Hamburg, c/o DESY, Building 25A, Notkestraße 85, 22603 Hamburg, Germany

Correction to: *Scientific Reports* 10.1038/s41598-019-52114-8, published online 28 November 2019

The original version of this Article contained a typographical error in the spelling of the author Vladimír Kopecký Jr., which was incorrectly given as Vladimír Kopecký. This has now been corrected in the PDF and HTML versions of the Article. The Supplementary Information was correct at time of publication.

