# Supplementary material for: Production of recombinant soluble dimeric C-type lectin-like receptors of rat natural killer cells
Source: Sci Rep. 2019 Nov 28;9:17836. doi: 10.1038/s41598-019-52114-8 (PMC6882821; doi:10.1038/s41598-019-52114-8)
Supplement: Supplementary file 1 — Supplementary Information [file 41598_2019_52114_MOESM1_ESM.pdf]

Supplementary Information

Production of recombinant soluble dimeric C-type lectin-like receptors of rat natural killer cells

Ondřej Vaněk[1*], Petra Celadova[1], Ondřej Skořepa[1], Jan Bláha[1#], Barbora Kalousková[1], Anna Dvorská[1], Edita Poláchová[1], Helena Pucholtová[1], Daniel Kavan[1,2], Petr Pompach[2], Kateřina Hofbauerová[2,3], Vladimír Kopecký Jr.[3], Aruz Mesci[4], Sebastian Voigt[5], James R. Carlyle[4]

[1] *Department of Biochemistry, Faculty of Science, Charles University, Hlavova 2030/8, 12840 Prague, Czech Republic*

[2] *Institute of Microbiology, The Czech Academy of Sciences, Vídeňská 1083, 14220 Prague, Czech Republic*

[3] *Institute of Physics, Faculty of Mathematics and Physics, Charles University, Ke Karlovu 5, 12116 Prague, Czech Republic*

[4] *Department of Immunology, University of Toronto, 1 King's College Circle, M5S 1A8 Toronto, ON, Canada*

[5] *Department of Infectious Diseases, Robert Koch Institute, Seestraße 10, 13353 Berlin, Germany*

[*] Corresponding author. E-mail address: ondrej.vanek@natur.cuni.cz

[#] *Current address: EMBL Hamburg, c/o DESY, Building 25A, Notkestraße 85, 22603 Hamburg, Germany*

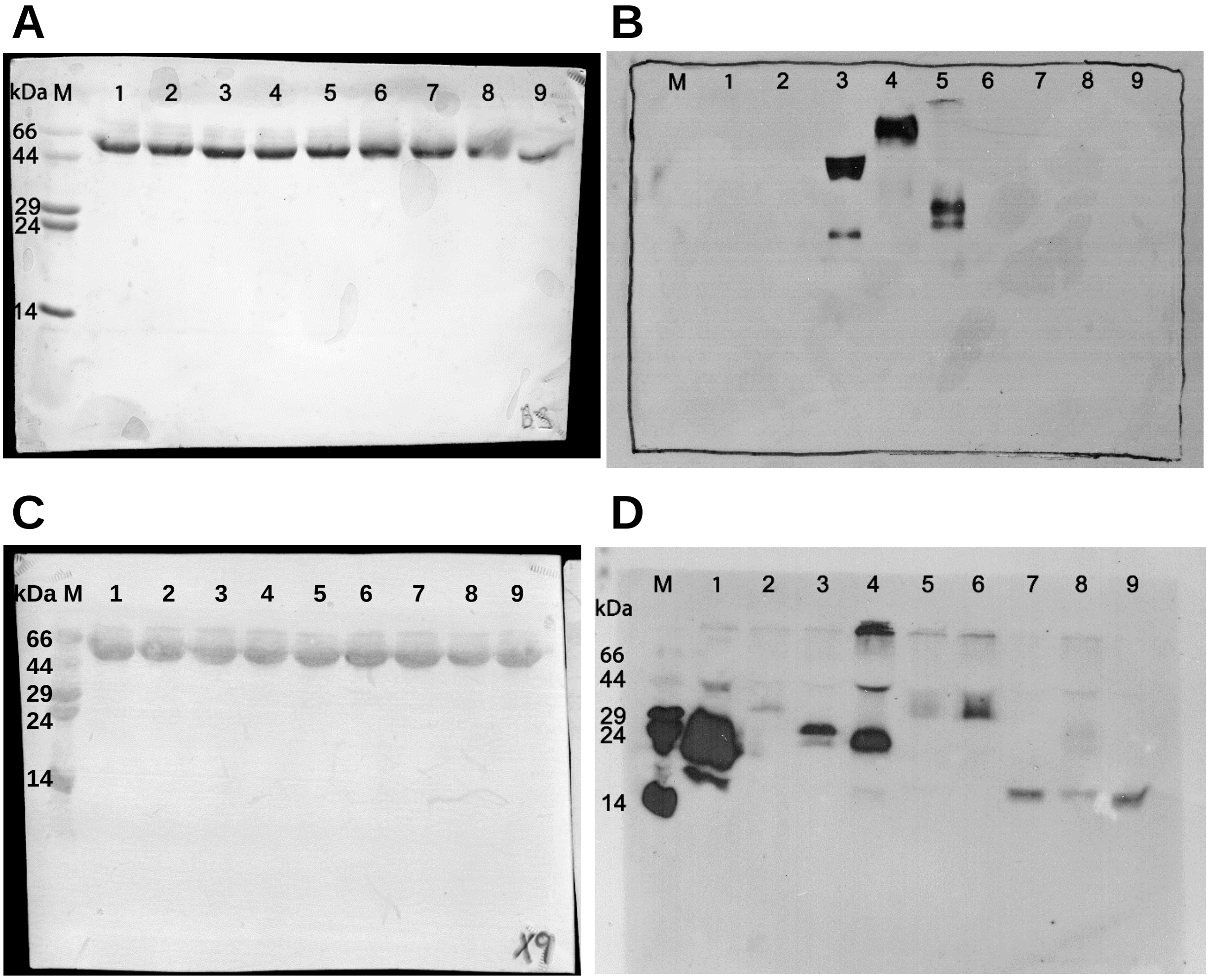

Figure S1. Full-size images of membranes and photographs used to analyse the small-scale expression test of pHLsec constructs in Figure 3A. Samples of transfected cell culture supernatants were resolved by 15% SDS-PAGE under non-reducing conditions, transferred onto nitrocellulose membranes, and transferred proteins were first stained with Ponceau Red (A and C) and then proteins containing histidine tag were detected by primary PentaHis mAb and secondary horseradish peroxidase-conjugated goat anti-mouse IgG polyclonal antibodies, and peroxidase activity was visualized by luminol chemiluminescence captured on photographic films (B and D). Lanes: M, marker; 1, mock transfected; 2, RCTL I38-T180; 3, Clr-11; 4, NKR-P1B^{WAG}; 5, NKR-P1B^{SD} (A and B); M, marker; 5, RCTL H51-T180; 6, RCTL H51-T170 (C and D); other lanes contain unrelated samples of supernatants resulting from expression tests of proteins that are not subject of the present study.

Table S1. Sequences of primers used for PCR amplification of given protein expression constructs and of vector-specific primers used for sequencing. AgeI/KpnI restriction cloning sites are highlighted in bold and underlined.

Target	AA	DNA primer
**Clr-11^{WAG}**	V65	5’-AAAAAA**ACCGGT**GTAAAAATGACACCACAGATCTCA-3’
	M207	5’-AAAAAA**GGTACC**CATAGGAGAAAAAGGAGTTTTGCA-3’
**NKR-P1B^{WAG}**	V78	5’-AAAAAA**ACCGGT**GTTCAAGAGAACAGGACAAAAACA-3’
	S223	5’-AAAAAA**GGTACC**GGAGCCATTACACATGCATTCACA-3’
**NKR-P1B^{SD}**	V78	5’-AAAAAA**ACCGGT**GTTCAAGAGAACAGGACAAAAACA-3’
	S223	5’-AAAAAA**GGTACC**GGAGTCATTGCACGTGCTTTC-3’
**pHLsec**	FW	5’-GCTGGTTGTTGTGCTGTCTCATC-3’
**pHLsec-FcHis**	REV	5’-CACCAGCCACCACCTTCTGATAG-3’
**pYD5**	FW	5’-TGATATTCACCTGGCCCGATCTG-3’
	REV	5’-TATGTCCTTCCGAGTGAGAG-3’
